# The molecular dynamics of bacterial spore and the role of calcium dipicolinate in core properties at the sub-nanosecond time-scale

**DOI:** 10.1038/s41598-020-65093-y

**Published:** 2020-05-19

**Authors:** Alexandre Colas de la Noue, Francesca Natali, Fatima Fekraoui, Patrick Gervais, Nicolas Martinez, Jean-Marie Perrier-Cornet, Judith Peters

**Affiliations:** 1 AgroSup Dijon, Université de Bourgogne-Franche-Comté, UMR 02.102 PAM, 1 esplanade Erasme, 21000 Dijon, France; 2 CNR-IOM, OGG, 71 avenue des Martyrs, CS 20156, 38042 Grenoble, Cedex 9 France; 3 Institut Laue-Langevin, 71 avenue des Martyrs, CS 20156, 38042 Grenoble, Cedex 9 France; 4 Univ. Grenoble Alpes, CEA, CNRS, IBS, 38000 Grenoble, France; 5 Univ. Grenoble Alpes, CNRS, LiPhy, 38000 Grenoble, France; 6 Qualisud, Univ Montpellier, CIRAD, Montpellier SupAgro, Univ d’Avignon, Univ de la Réunion, Montpellier, France

**Keywords:** Biophysics, Microbiology, Biological physics

## Abstract

Bacterial spores are among the most resistant forms of life on Earth. Their exceptional resistance properties rely on various strategies, among them the core singular structure, organization and hydration. By using elastic incoherent neutron scattering, we probed the dynamics of *Bacillus subtilis* spores to determine whether core macromolecular motions at the sub-nanosecond timescale could also contribute to their resistance to physical stresses. In addition, in order to better specify the role of the various spore components, we used different mutants lacking essential structure such as the coat (PS4150 mutant), or the calcium dipicolinic acid complex (CaDPA) located in the core (FB122 mutant). PS4150 allows to better probe the core’s dynamics, as proteins of the coat represent an important part of spore proteins, and FB122 gives information about the role of the large CaDPA depot for the mobility of core’s components. We show that core’s macromolecular mobility is not particularly constrained at the sub-nanosecond timescale in spite of its low water content as some dynamical characteristics as force constants are very close to those of vegetative bacteria such as *Escherichia coli* or to those of fully hydrated proteins. Although the force constants of the coatless mutant are similar to the wild-type’s ones, it has lower mean square displacements (MSDs) at high Q showing that core macromolecules are somewhat more constrained than the rest of spore components. However, no behavior reflecting the glassy state regularly evoked in the literature could be drawn from our data. As hydration and macromolecules’ mobility are highly correlated, the previous assumption, that core low water content might explain spores’ exceptional resistance properties seems unlikely. Thus, we confirm recent theories, suggesting that core water is mostly as free as bulk water and proteins/macromolecules are fully hydrated. The germination of spores leads to a much less stable system with a force constant of 0.1 N/m and MSDs ~2.5 times higher at low Q than in the dormant state. DPA has also an influence on core mobility with a slightly lower force constant for the DPA-less mutant than for the wild-type, and MSDs that are ~ 1.8 times higher on average than for the wild-type at low Q. At high Q, germinated and DPA-less spores were very similar to the wild-type ones, showing that DPA and core compact structure might influence large amplitude motions rather than local dynamics of macromolecules.

## Introduction

When deprived of nutrients, *Bacillus* and *Clostridia* bacteria can form endospores which are metabolically inactive and they can remain in this dormant state for years. Besides their ability to survive to starvation, bacterial spores are also highly resistant to various stresses such as heat, radiation or toxic chemicals^1^. Because of these exceptional properties, they are involved in food spoilage, foodborne diseases, they could be a threat as biological weapons, and might represent a probable vehicle for transfer of life between planets^2,3^. From the most external layers, bacterial spores (oval endospores of 1–1.5 μm diameter) are composed of an exosporium (absent in *Bacillus subtilis*), two proteinaceous layers called the inner and outer coat, the outer membrane and the cortex, which is mainly composed of a thick peptidoglycan structure, a cell wall, and the inner membrane, which is surrounding the protoplasm or core^1^. The latter contains the genetic material, protected by chaperone proteins called small acid soluble spore proteins (SASPs), mainly involved in resistance to wet heat and ultraviolet radiations^2,4^. The core is characterized by a low water content (0.6 *h*, where *h* = g_water_/g_dry protein_), an elevated concentration of ions and a complex of dipicolinic acid (DPA) and Ca^2+^. This complex can represent up to 20% of spore core dry weight^5^ and is involved in the wet heat resistance of the spores. Their multi-resistance is the consequence of various protective strategies involving, but not limited to, (i) the detoxication through external layers of the spore (i.e. coat)^6,7^, (ii) a reduced permeability and mobility of the inner membrane^8,9^, and (iii) the protection of core content through a restriction of the macromolecules’ mobility. First experiments using Nuclear Magnetic Resonance (NMR) or differential scanning calorimetry (DSC) showed a low mobility of bacterial spore, and a transition was identified as a potential glassy state originally attributed to the core^10,11^. However, it was challenged later, because these observations might rather be interpreted as a denaturation of coat proteins^12^. Further experiments with electron spin resonance (ESR) allowed to measure the high microviscosity of *Bacillus* spore core and have shown that the microviscosity is much higher in the core of dormant spores than in germinated spores^13^. Using a cytoplasmic GFP (Green Fluorescent Protein) fusion, Cowan and co-workers demonstrated with FRAP (Fluorescence Recovery After Photobleaching) method that the proteins in the core are immobile over a 10 second time course and have a diffusion coefficient ≈ 3 order of magnitude slower than for a protein in solution of similar size^14^. Confocal microscopy and Raman micro-spectroscopy analysis has shown in addition that water molecules in the spores were in a weak hydrogen bonded mode as compared to the strong hydrogen bonded state in pure water. Moreover, spatial distribution of water was found to be less dense in the core than in other spore regions and distinct from the DPA and protein region^15^. Another publication suggests that DPA may be at least partially in an amorphous solid-like environment state^10^, even if water mobility within the core indicates that some of the DPA molecules should be bound to water^15^.

Recently, the physical properties of water in bacterial spores have been investigated by NMR^4^ and dielectric spectroscopy^11^. Both studies found that water mobility was quite similar to what was found in a binary protein water system at the same hydration level, even in the dense core^16^, and that the majority of intracellular water was indistinguishable from bulk water^11^. Therefore, the state of water alone does not explain the extreme heat resistance of bacterial spores. It has been suggested that the dormancy and the resistance was not due to a quenching of molecular diffusion, but rather to an immobilization of protein rotation, probably as a consequence of the low core hydration^5^.

During germination this mobility will be increased in relation with rehydration of core. Germination is generally decomposed in 2 stages: first stage which is essentially passive and results in a partial rehydration of spore core and a massive release of CaDPA and ions and the second stage which consists in coat and cortex lysis and the full rehydration of spore core. It has been shown that the full germination has a great impact on protein mobility while stage I of germination has only few impact on motility and core microviscosity which remain comparable to that of the dormant spore although most of DPA left^13,14^.

Neutron scattering is a useful tool for investigating the properties of biological materials and water inside^17^ as the technique is non-destructive, and due to their lack of electrical charges neutrons can deeply penetrate into matter. Hydrogen atoms largely dominate the incoherent neutron scattering cross section and are homogeneously distributed in biological macromolecules, providing thus a perfect tool to explore molecular dynamics. The energy resolution of neutron spectrometers allows monitoring molecular motions from picosecond to nanosecond time scales. From elastic measurements, it is possible to extract the atomic mean square displacements (MSD) and the effective force constant that are a measure of protein flexibility and stability, respectively^18^. In the past decade, it was mainly used to characterize the dynamics of various protein systems and their surrounding water^19–21^, but systems that are more complex are also progressively investigated. Tehei and co-workers^22^ showed through an *in vivo* study that psychrophilic, mesophilic and thermophilic bacteria might adapt the proteins flexibility to their environment. Recently, incoherent neutron scattering was successfully used to characterize animal proteins^23^, but also whole cells^24^, biological tissue^25^ and biological nanoparticles^26^.

In a previous paper we reported a first successful study of the spore dynamics with neutrons, in which we used the backscattering spectrometer IN13^27^ at the Institut Laue Langevin (ILL) in Grenoble/France to investigate *Bacillus subtilis* spore powder, hydrated at a level of 0.4 *h*^28^. We found some similarities with earlier DSC measurements regarding the phase transitions ^12,29,30^, but interestingly, the spores did not undergo the dynamical transition observed for proteins at ~240 K at the same hydration level. The IN13 instrumental resolution of 8 μeV gives access to internal motions within the timescale up to 100 ps, that are dominated by the dynamics of proteins and possibly by confined water^31^. We tried here to isolate the signal from the proteins from the signal of the spore core by using mutants, one lacking most of the proteins of the coat (PS4150) and the other lacking a major part of the large DPA depots present in the wild type spore (FB122). Furthermore, we studied the dynamics of germinated spores by comparing the wild type to a mutant lacking the major cortex lytic enzyme (FB113). This specificity allows the blocking of the germination process at an intermediate stage (stage I), where DPA is released and water penetrates the core, but the cortex is not hydrolyzed^32^.

The main motivation of the present study is therefore to investigate the dynamical properties of bacterial spores and more specifically of the core using elastic incoherent neutron scattering (EINS) measurements. Such investigation will permit to shed light on spore components’ mobility, in an effort to relate it to the known properties of the spore’s water mobility, and its uncommon resistance properties.

## Materials and Methods

### Strains

**Figure 1:**
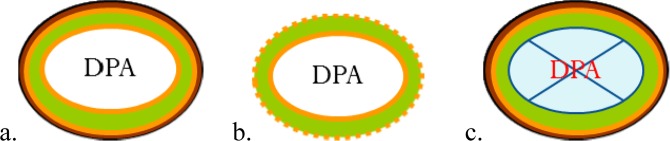
Schematic representation of the four strains used in this study. (**a**) PS533 (wild-type), (**b**) Coat-less PS4150 (Δ*cotE::tet* Δ*gerE::spc*), (**c**). DPA-less FB122 (Δ*spoVF::tet* Δ*sleB::spc*). The brown layer represents the inner and outer coat, the orange layer the outer membrane, the green layer the cortex, the second orange layer the cell membrane and inner membrane. The core contains DPA, if not deleted. Strain FB113 (Δ*cwlJ::tet* Δ*sleB::spc)* in the dormant form has similar structure organization as PS533 (**a**), and when germinated might be structurally comparable to FB122 (**c**).

Strains main features are depicted in Fig. 1. Strain PS533 is the wild type strain and carries a plasmid pUB110 encoding resistance to kanamycin. It is an isogenic derivative of the strain PS832. Strain PS4150 is deleted for *cotE* and *gerE* coding sequences, and lacks most inner and outer proteinaceous coat layers^33^. Strain FB122 is deleted for the *spoVF* operon, leading to a spore lacking most part of the large DPA depots present in the core (<2% of the DPA of the wild type), and it is stabilized through the deletion of *sleB* genes that codes for the enzymes responsible for the lysis of the cortex^34,35^. FB113 carries mutations on *cwlJ* and *sleB* genes encoding the two cortex lytic enzymes that allow the hydrolysis of the cortex during germination. FB113 releases most of the DPA during germination, and water penetrates the core, but it is not able to degrade its cortex, leading to a partially germinated spore with an intact cortex. As a consequence, the core wet density remains higher for FB113 germinated spores than for the germinated wild-type, where cortex has been hydrolyzed^32^.

### Spore production

Three protocols were employed for spore production. For most samples, 2xSchaeffer’s medium-glucose (2xSG) was used, either on agar plate (called 2*SG agar hereafter) or liquid medium (called 2*SG liquid)^36^. Alternatively, we used Spizizen minimal medium with 0.1% casamino acid agar plates (called SPZ agar), as described elsewhere^37,38^. It was used as an alternative, because spore of PS4150 mutant strain was much more stable and easier to produce on this medium, particularly for preparations in suspensions. All spore preparations were produced at 37 °C for 48 to 72 h before harvesting. Spore suspensions were washed at least 10 times with cold distilled water prior to use, stored for at least one week at 4 °C and washed again several times. All spore preparations were exempt of spore debris and germinated cells and purity was higher than 98% as observed by phase-contrast microscopy.

To avoid potential differences originating from the sporulation on different media, comparisons were performed essentially for strains produced in exactly the same conditions (SG agar, SG liquid or Spizizen agar).

Spores were germinated for 1 hour at a concentration corresponding to an optical density at 600 nm (OD_600_) value of 2 in tris-HCl pH 8 supplemented with L-valine 10 mM to trigger germination. Beforehand the spores were activated at 70 °C-30 min at a high concentration (OD_600_ = 10) to increase and synchronize spores’ germination response^39^. Then the spores were recovered by centrifugation at 10000 g -5 min to pellet cells and prepared for neutron experiments as explained hereafter.

### Spore preparation for neutron experiments

Spore samples were prepared in D_2_O or H_2_O 5 mM phosphate buffer at pH 8 (not corrected for isotope effects). Spores in deuterated water buffer were washed twice with 40 ml of deuterated buffer to remove most of the water that is freely exchanged in the spore, and were then kept in deuterated buffer at 10 °C for at least 8 hours to exchange the water that could remain in the core. Core water is supposed to freely exchange with D_2_O in the time-lapse considered here as already supported by many authors^5,15,40^. For germination experiments, the germinated spores were washed three times with D_2_O phosphate buffer pH 8 to eliminate residuals of H_2_O and DPA. For samples in the powder form, spores in H_2_O were freeze-dried for 48 hours and further dehydrated in a closed desiccator over a saturated salt solution of lithium bromide in D_2_O. Afterward, spores were weighed directly in the sample holder and placed in a chamber containing a saturated salt solution of KCl in D_2_O (RH ~85%), if necessary transferred into a chamber with pure D_2_O and kept until an hydration of ~ 0.4 *h* was obtained.

A summary of sample characteristics is given in Table 1 regarding the medium used for preparation, the hydration level *h*, stability during neutron scattering measurement, core wet density and measurement time per temperature point. After the neutron experiment, a part of the sample was used to determine the dry matter content (for spores’ suspension and pellets), and the other part was suspended in distilled water to gain information about the stability during neutron scattering measurement. The samples were checked by phase contrast microscopy in order to observe their phase-bright properties, and the resuspension solution was assessed for DPA level using the Tb^3+^ method described by Hindle and Hall^41^ or by measuring supernatant absorbance at 270 nm^42^.

**Table 1:** Main characteristics of the spore samples used during the neutron experiment and their preparation.

Sample preparation	Strains/ sporulation medium	Hydration level *h* (*h* = g water/g dry mass)	Stability during neutron measurement	Core wet density values in water (g/ml)	Measurement time per T point (h)
D_2_O pellets	PS 533 2*SG agar	1.2	Yes	1.353–1.382^30,32,69^	2.6
	PS 533 2*SG agar (germinated)	2.6	Yes	1.194^32^	3
	FB113 2*SG agar	1.1	Yes	1.351^32^	4
	FB113 2*SG agar (germinated)	1.57	Yes	1.25^32^	3
	FB122 2*SG agar	1.73	Yes	1.297^30^	2.7
H_2_O pellets	PS533 2*SG liquid	1.3	No	1.353–1.382^30,32,69^	2.3
	FB122 2*SG liquid	1.6	Yes	1.297^30^	2.3
D_2_O hydrated powder	PS533 2*SG liquid	0.36	Yes	1.353–1.382^30,32,69^	1.5-3 (according to T range)
	PS4150 2*SG liquid	0.39	Yes	1.376^29^	
	FB122 2*SG agar	0.4	Yes	1.297^30^	1-1.3
D_2_O suspensions	PS533 2*SG liquid	7	No	1.353–1.382^30,32,69^	3.5
	PS533 2*SG agar	6	Yes	1.353–1.382^30,32,69^	3.5
	PS533 SPZ agar	5.5	Yes	1.353–1.382^30,32,69^	3
	PS4150 SPZ agar	4.7	No	1.376^29^	3
H_2_O suspensions	PS533 SPZ agar	2.7	Yes	1.353–1.382^30,32,69^	2.5 h

### Elastic measurements on IN13

EINS measurements as a function of temperature were performed on the thermal (λ = 2.23 Å) high-energy resolution backscattering spectrometer IN13 (ILL, Grenoble, France)^27^, characterized by a very large momentum transfer range (0.3 < Q < 4.9 Å^−1^) between the incoming and scattered neutron with a good and nearly Q-independent energy resolution (8 μeV FWHM). IN13, therefore, allows accessing the space and time windows of 1.3–21 Å and 0.1 ns, respectively. The ILL data is available at 10.5291/ILL-DATA.8-04-668^43^, 10.5291/ILL-DATA.8-04-686^44^ and 10.5291/ILL-DATA.8-04-827^45^.

The elastic scattering intensities (I_el_(Q) = S(Q, ω ≈ 0)), properly corrected for the empty sample holder signal, were normalized with respect to a vanadium scan (typically used as a standard), to compensate for spurious background contributions and detector efficiency. The sample mass and thickness were suitably chosen to optimize the compromise between good signal-to-noise ratio and minimum multiple scattering contribution. For this purpose, a transmission above 90% was kept for all samples. Absorption correction was based on the correction formula of Paalman-Pings coefficients^46^ and performed using the ILL program LAMP^47^. For data analysis, we summed the intensities over the whole range of available scattering angles and calculated relative summed intensities by dividing by the lowest temperature point and multiplying by 100 to get it in %. Furthermore, atomic mean square displacements <u^2^ > (MSDs) were extracted from the slope of the elastic intensities following the approximate scattering law: I ~ I_0_ exp(−<u^2^ > *Q^2^/6), which is valid in the range of small Q satisfying the Gaussian approximation^48^ or slightly beyond^49^. The Q values are related to fluctuations according to Heisenberg’s uncertainty principle, e.g. small Q values are equivalent to large fluctuations and vice versa. Here we used as Q-range for all fits both ranges 0.5–1.67 Å^−1^ (called “low Q” hereafter) and 1.4–2.02 Å^−1^ (called “high Q” hereafter). The smallest Q-value of 0.3 Å^−1^ was excluded as the corresponding intensity contains information on larger motions not considered here. Such differentiation permits to focus selectively at low Q on larger fluctuations^44^ arising from diffusive motions of smaller molecules as the DPA or the solvent and at high Q on smaller macromolecular internal motions in the spore^20^. The MSD are a measure of the sample’s flexibility at a given temperature. Based on the temperature dependence of the MSD values, information about the resilience (stiffness) of the sample in a given hydration state and temperature domain can be obtained. The effective force constant, <k > (for temperatures where molecular motions are considered as anharmonic) in the samples can be calculated according to^18^ as

1 $$\langle k\rangle =\frac{0.00276}{d\langle {u}^{2}\rangle /dT}.$$

Here <k> is expressed in Newton per meter when <u^2^ > is given in Ångstrom squared and T is the absolute temperature.

MSD ratio was calculated in the stable temperature range (no germination). For that, the MSD of the mutant was divided by the MSD of the wild-type at the same temperature and averaged over all temperatures (Table 2).

**Table 2:** MSDs’ ratio average between mutants and wild-type in a temperature range excluding germination effects. All the ratios were calculated for spore samples produced on the same medium and probed by neutron scattering in similar buffer conditions.

Strains	Low Q	High Q
**MSD ratio**		
FB113ger/PS533	1.3 ± 0.2	1.2 ± 0.2
PS533ger/PS533	2.5 ± 0.3	1.3 ± 0.2
FB122_DPAless_/PS533	1.8 ± 0.1	1.1 ± 0.2
FB122_DPAless_/PS533 (in H_2_O)	1.3 ± 0.1	1.1 ± 0.2
PS4150_Coatless_/PS533	1.0 ± 0.1	0.6 ± 0.1
PS4150_Coatless_/PS533 (powder 0.4 *h*)	0.9 ± 0.1	0.7 ± 0.1

## Results

To get an exhaustive picture of the molecular dynamics of spore molecules and the role of the water on it, we investigated the samples as function of temperature in various hydration conditions and grown in different sporulation media.

### Influence of the sporulation medium on the dynamics of the spores

**Figure 2:**
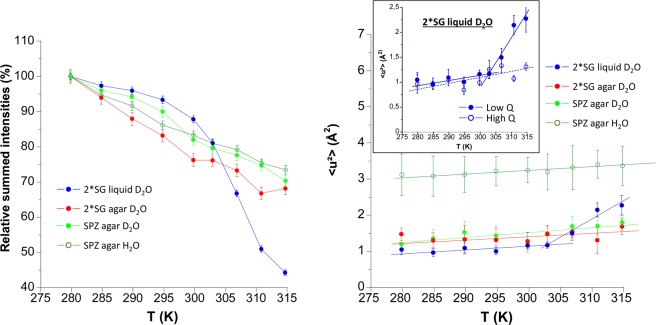
Temperature dependence of the relative integrated elastic intensities (left side) and MSD extracted from the low Q range (right side) of the WT PS533 in H_2_O or D_2_O suspension produced with different sporulation media: 2*SG liquid (filled blue circles), 2*SG agar (filled red circles), and Spz agar in H_2_O (open dark green circles) and D_2_O (in filled light green circles). Insert on the right shows the absence of transition at high Q (empty blue circles) during germination process, as compared to the large transition observed at low Q (filled blue circle). The straight lines on the right side are linear fits to extract the effective force constants (see Table 3).

Figure 2 (left side) shows relative summed intensities of wild type spores, all with a high hydration level >4.5 *h* (see Table 1), grown on Spz agar (green symbols), 2*SG agar (red symbols), and 2*SG liquid (blue symbols). For the sake of beam time limitation, we compared only one sample in suspension, hydrated in H_2_O (open circles) and in D_2_O (filled circles), grown on Spz agar. The hydration level in H_2_O was, however, lower to avoid absorption effects, as the neutron scattering cross section of hydrogen atoms is much larger than that of its isotope deuterium, but we do not expect effects on the macromolecular dynamics at such degree of hydration. Anyhow, no isotope effect could be identified, i.e. the relative summed intensities in H_2_O or D_2_O were almost identical in slope within error bars.

All samples were probed with very similar scanning times, i.e. 3.5 h/T point for the samples grown on 2*SG agar and 2*SG liquid and 3 h/T point for the sample grown on Spz agar (see Table 1). Although the media of 2*SG agar and Spz agar result in very similar sample kinetics with summed intensities almost linearly decreasing with temperature, the 2*SG liquid medium gave rise to a fast drop of the summed intensities above 300 K reflecting an important change in the dynamics of the sample. Phase contrast check after the end of the neutron scattering experiment and further DPA assay on the recovered sample confirmed that most of the spores had spontaneously germinated, and thus changed their state. There could be another change of slope above 315 K probably indicating the end of the germination process, but due to instrumental limitations, we could not reach higher temperature.

The MSDs extracted from the low Q range (Fig. 2, right side) are much higher for the sample in H_2_O than for all other samples, which present very similar MSDs within error bars. It can be hypothesized that such finding is due to the fact that in D_2_O, hydrogen atoms of the spore components or the water confined within the spore are exchanged against deuterium atoms and are or no longer visible in incoherent neutron scattering in contrast to a H_2_O solution, where all these scatterers fully contribute.

We show furthermore in the Electronic Supplementary Information (ESI) figures of the MSDs extracted from the two Q ranges (Fig. S1) and an example of the fit of ln(I/I_0_) vs. Q^2^ (Fig. S2) and find that the curves corresponding to the samples grown on Spz agar in light and heavy water are almost superposed in the high Q range, but affected with larger error bars than in the low Q region, as neutron intensities drop as Q increases. Internal motions are thus not affected by the solution composition, while the MSDs at low Q are higher in H-buffer, because of the motion of the H atoms belonging to the confined solvent and labile hydrogen participating to the signal.

Another important finding is that the spontaneous germination observed through the sharp decrease in intensity was also very well resolved on the MSD values at low Q (Fig. 2, right panel). However, this phenomenon was completely absent from the MSDs extracted at high Q (see insert, right panel, Fig. 2).

### Influence of the coat on the dynamics of the spores

Coatless PS4150 and WT PS533 were studied with two different spores’ preparations (2*SGliquid and SPZagar), as hydrated powder, and as suspension.

**Figure 3:**
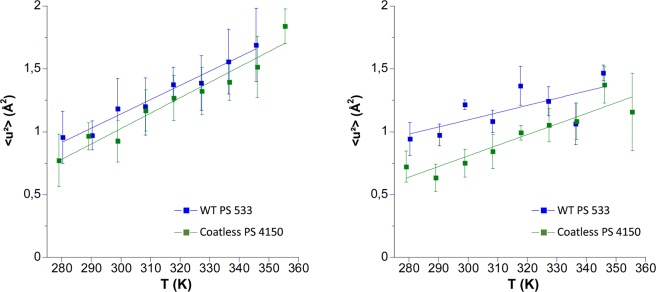
MSD extracted from the low (left) and high (right) Q range of powder samples hydrated at ~0.4 *h* in D_2_O (see Table 1) of the WT PS533 and Coatless PS4150. Spores were produced in 2*SG liquid medium, freeze-dried and rehydrated by D_2_O sorption in closed chamber until a hydration of ~0.4 *h* was obtained. The linear curves are fits to extract the effective force constants k’ (see Table 3).

Figure 3 illustrates the MSDs extracted at low and high Q-range of the D_2_O hydrated powder of WT PS533 and coatless PS4150 (see Table 1). This hydration level is commonly accepted as to correspond to about one water layer around a globular protein^50^, permitting to study the dynamics of a biological system in a sufficiently wet state to allow functionality. The straight lines are linear fits to the curves, which permit to extract the effective force constants according to Eq. (1) (see Table 3).

**Table 3:** Summary of samples’ effective forces constants as determined by fitting MSDs. Effective force constants were determined at low Q and high Q ranges as specified. If not indicated otherwise, the force constants were extracted from the whole temperature range.

Sample preparation	Strains/sporulation medium	Effective force constant k’ (N/m) Low Q (0.5 < Q < 1.67 Å-1)	Effective force constant k’ (N/m) High Q (1.4 < Q < 2.0 Å-1)
D_2_O pellets	PS 533 2*SG agar	0.40 ± 0.11	0.21 ± 0.05
	PS 533 2*SG agar (germinated)	0.10 ± 0.02	0.44 ± 0.35
	FB113 2*SG agar	0.38 ± 0.10	0.23 ± 0.05
	FB113 2*SG agar (germinated)	0.26 ± 0.10	0.26 ± 0.09
	FB122 2*SG agar	0.24 ± 0.04	0.19 ± 0.05
H_2_O pellets	PS533 2*SG liquid	[280–303 K] 0.38 ± 0.14	11 ± 80**
		[303–315 K] 0.05 ± 0.01*	
	FB122 2*SG liquid	0.31 ± 0.04	0.47 ± 0.23
D_2_O hydrated powder	PS533 2*SG liquid	0.25 ± 0.02	0.46 ± 0.15
	PS4150 2*SG liquid	0.23 ± 0.02	0.29 ± 0.04
D_2_O suspensions	PS533 2*SG liquid	[285–307 K] 0.27 ± 0.11	0.37 ± 0.17
		[307–315 K] 0.028 ± 0.010*	
	PS533 2*SG agar	0.29 ± 0.13	0.38 ± 0.15
	PS533 SPZ agar	0.20 ± 0.05	0.24 ± 0.2
	PS4150 SPZ agar	[280–307 K] 0.25 ± 0.16	1.6 ± 1.6**
		[307–315 K] 0.010 ± 0.003*	
H_2_O suspensions	PS533 SPZ agar	0.31 ± 0.04	0.53 ± 0.75

*Calculations of k’ during germination has no physical meaning as the sample is evolving throughout this process but it allows to visualize the large increase in motions amplitude occurring during germination.

**Statistics was not sufficient to perform acceptable fits; as the slope is almost zero, the calculated effective force constant becomes meaningless.

The MSDs reach higher values at high temperature in the low Q range, because they correspond to larger amplitudes. The slopes of both samples are very similar, with k’ of 0.23 and 0.25 N/m for PS4150 and PS533, respectively (Table 3), which is indicative of almost identical types of motions at low Q. However, if constant forces were quite similar for both Q ranges, MSDs at high Q were lower for the coatless PS4150 than for the wild-type as shown by the MSD ratio (Table 2).

**Figure 4:**
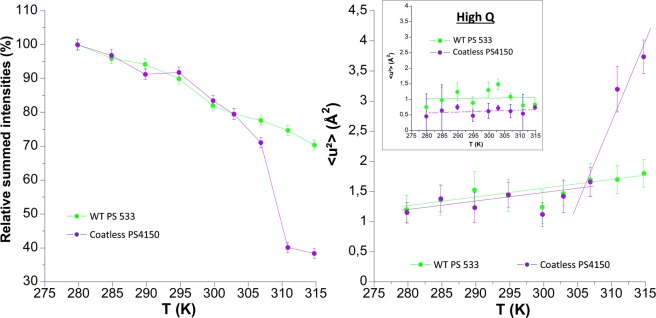
Temperature dependence of the relative summed intensities (left sight) and MSDs (right side) of spores produced on SPZ agar medium in D_2_O suspension: WT PS533 (green) and Coatless PS4150 (purple) spores. MSDs are extracted at low Q, and insert show the MSDs extracted at high Q where germination has no effect. The straight lines on the right side are linear fits to extract the effective force constants (see Table 3).

In a next step, we compared the wild type sample with the PS4150 strain, but in D_2_O suspensions, (see Table 1) in order to ensure a full mobility of all spore components that might be hampered in the hydrated powder. Both samples were grown on Spz agar, and the temperature was increased with a slow ramp of 3 h/T point (see Fig. 4). By contrast to the wild type, a drastic drop of the relative summed intensities was observed for the coatless PS4150 mutant, that has been related to spontaneous germination above 305 K (loss of spore refractility and DPA leakage >95%). The slopes of (k’) are the same for both samples at low and high Q, but the increase in MSDs due to germination is only visible at low Q for larger motions. As observed for hydrated spore powders, MSDs absolute values of the coatless PS4150 mutant and the wild type were comparable in the temperature range between 280 and 307 K at low Q, but were lower for the mutant in the high Q range. This indicates that local motions in coatless spores might be more constrained than in the wild type.

### Influence of DPA and core compact structure on the dynamics of the spore

**Figure 5:**
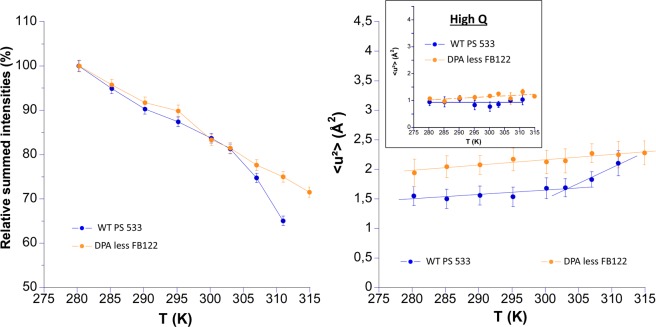
Relative summed intensities (left side) and MSDs extracted at low Q (right side) of wild type (blue) and DPA-less mutant (orange). Spores were produced on 2*SG-liquid medium and prepared in H_2_O buffer as pellets. Insert on the right side show the MSDs extracted at high Q where germination has no effect. The straight lines on the right side are linear fits to extract the effective force constants (see Table 3).

**Figure 6:**
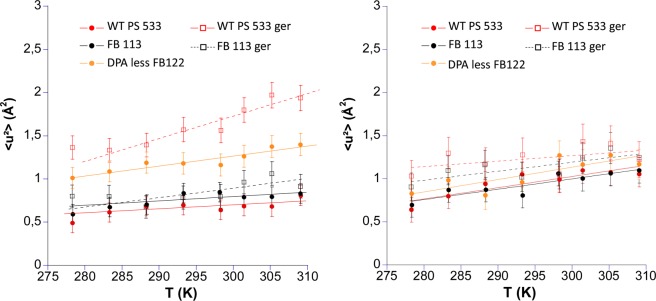
MSDs of the wild type PS533 (red) in the dormant form (filled circles) and after germination (empty squares), FB113 mutant (black) in the dormant form (filled circles) and after incomplete germination (empty squares), and of DPA-less FB122 (orange filled circles). Spores were produced on 2*SG agar medium and samples were prepared in D_2_O buffer as pellets. The left panel corresponds to the low Q range and the right panel to the high Q range. The straight lines are linear fits to extract the effective force constants (see Table 3).

To evaluate the influence of DPA and of the core compact structure on the dynamics, DPA less spore (FB122), CLE enzyme deficient spore (FB113) which can produce partially germinated spore (FB113ger) and WT fully germinated spore (PS533ger) were compared to WT dormant spores (PS533). Spores of FB122 and PS533 were produced in 2*SG liquid medium and analyzed in H_2_O buffer (Fig. 5). In addition, wild type, FB122 and FB113 spores were prepared on 2*SG agar plates and measurements were made in D_2_O buffer as pellets (Fig. 6).

As observed earlier for PS533 produced on 2*SG liquid medium in D_2_O buffer (see Fig. 2), the intensity started to drop for spores produced in the same conditions, but probed in H_2_O buffer (Fig. 5). It is clearly correlated to the germination process, as confirmed by phase contrast microscopy and DPA release measurement (data not shown). In contrast, the DPA-less mutant FB122 does not germinate during the whole experiment, whether being produced on 2*SG agar or 2* SG liquid and in both D_2_O and H_2_O (Figs. 5 and 6). This was not surprising as DPA is essential for the activation of the remaining CwlJ cortex lytic enzymes, thus FB122 germinates very poorly, even when germination is triggered by nutrients^51^.

In H_2_O and D_2_O buffer, MSDs of the DPA-less mutant FB122 were slightly above those of the wild type PS533 at high Q (Fig. 5, insert in right panel), but still within the error range, whereas they were markedly higher at low Q (Fig. 6, left panel). The difference can be explained by a higher resolution of WT PS533 spore component mobility in D_2_O than in H-buffer where additional scatterers could contribute to the signal, particularly at low Q.

FB113 and PS533 in the dormant form were almost similar regarding their MSD values at low and high Q, confirming that the mutation leading to the absence of cortex lytic enzymes did not modify spores’ mean dynamics. Their force constants were comparable for both samples (Table 3).

Partially germinated spores FB113ger were compared to DPA-less FB122 because their core features should be comparable (no DPA, higher hydration with a wet density d ≈ 1.2–1.25 *vs* 1.35 for wild type dormant spore). Partially germinated FB113ger and DPA-less FB122 exhibited comparable force constants at both Q ranges (low Q ~ 0.24 –0.26 N.m^−1^ and high Q ~ 0.19–0.26 N.m^−1^) ranges showing a more homogeneous behavior regarding motions of small and higher amplitudes than for the WT PS533 and FB113.

Surprisingly, whereas partially germinated FB113ger exhibited force constant at low Q akin to DPA-less FB122, partially germinated FB113ger MSD absolute values remained very low as compared to DPA-less FB122 under similar conditions, much closer to the MSD values observed for the ungerminated wild-type. It could be hypothesized that the repartition of water within FB113 spore after incomplete germination and DPA release does not lead to the same structural organization inside the core than for DPA-less FB122, which accomplishes sporulation process without accumulating CaDPA in the core.

As expected, fully germinated spores (PS533ger) had the highest MSD values of all samples and the lowest force constant of (0.1 N.m^−1^) at low Q, highlighting the important structural changes undergone during germination.

Strikingly, force constants and MSDs of DPA less FB122, partially germinated spore FB113ger and even fully germinated spore PS533ger were very close to both wild-type PS533 and FB113 at high Q, whereas significant differences were noted at low Q for PS533ger and DPA-less FB122 as compared to the wild-types. As high Q range represents motions of small amplitudes (i.e. local motions), it means that core compact structure and components such as DPA have almost no or very small effect on the local dynamics of core’s macromolecules, but clearly impact the large amplitude motions observable at low Q.

## Discussion

### Spontaneous germination of the samples

The results presented in this study provide information about the potential of elastic incoherent neutron scattering to detect complex biological processes such as bacterial spore germination. Strikingly, the germination of the spores is accompanied by a sharp drop in neutron intensity and a large increase of <u²>, noticeable only in the small Q range. Such effect is due to a significant increase in hydrogen motions and therefore due to a drastic change of the macromolecular environment, probably of the core. The intensity drop is irreversible as shown in an earlier study by a measurement at lower temperature after the highest temperature was reached^28^ and confirmed in the present study. It was furthermore verified by our measurement on fully germinated spores (Fig. 6) for which the MSDs were clearly higher than for dormant spores at low Q. Overall, the various samples did not respond similarly to the temperature rising steps, depending on different factors: the production media, the structure of the sample (deletion of structural genes), but also the hydration level (hydrated powder at about 0.4 *h*, or liquid/pelleted spores).

To the best of our knowledge, the phenomenon of spontaneous germination along a slow temperature rise and its action on the molecular dynamics has not been described so far. According to Rose *et al*.^52^, most structures are identical for spores produced in liquid or on solid media. Here we established that the sporulation environment had a strong influence on the stability, as wild type spores PS533 produced on solid media seemed to be much more stable than the ones produced in liquid media. Indeed, some differences are known between the spores arising from different media, mostly concerning the inner membrane composition, for which the ratio of anteiso/isofatty acids is higher for spores produced on plates^52^. Thus plated spores may have a more fluid membrane with a lower melting point, because anteiso fatty acids induce a packing disruption and a higher degree of fluidity^53,54^. Whether the inner membrane fluidity of the spores played a pivotal role in the spontaneous germination process observed here cannot be directly determined within this study, but it opens the discussion on some interesting questions and perspectives. However, Rose *et al*.^52^ already reported some counterintuitive results with non-nutrient germination triggered by dodecylamine that acts directly on the inner membrane or spores’ DPA channel. Despite the previous observation that spores with higher membrane fluidity (produced at low temperature) germinated faster, and assuming that DPA could more easily cross the membrane when destabilized by dodecylamine^55^, plate produced spores germinated slower than did the liquid produced spores^52^.

These observations based on membrane composition should, however, be taken with caution, because the inner spore membrane cannot be considered as a typical bilayer membrane for which mobility depends on fatty acid composition and temperature, only. In fact, the inner membrane embedded inside the spore complex structure has been described as densely packed and largely immobile^9^ with a much higher viscosity than a “free” membrane of similar composition, as shown by the drastic drop of viscosity in germinated spores^8^. The proportion of immobile lipids in dormant spores is ≈ 70% whereas it drops to ≈25% in germinated spores (see Table 4), similar to the proportion observed in growing cells. Recently, it was shown that lipid droplets were stored as reservoirs for further membrane expansion during germination^56^.

**Table 4:** Relative volume of main spore layers and evolution of these structures after spore germination.

Spore layers	Dormant spore	Germinated spore
Coat	Relative V ~40% spore 25–50% of spore total proteins	Partial degradation/cracked
Cortex	Relative V~28%	Hydrolysed
Inner membrane	Relative V negligible ~70% Immobile lipids Low permeability	Expansion of inner membrane ~25% Immobile lipids Higher permeability
Core	Relative V~32% Ca-DPA~10% of spore and 20% core dry weight DNA Proteins (SASPs) Water 0,6 h	Leakage of the DPA Water uptake Protein hydrolysis

Aside eventual membrane structure variations, bacterial spores produced on plates also exhibit higher resistance to temperature, and a slower germination rate in response to nutrients. If Rose *et al*.^52^ report no significant variations in their coat composition, Abhyankar *et al*. recently showed that the coat of spores produced on plates was significantly different from the ones produced in liquid regarding proteins’ proportion between the inner coat and the outer coat, but also by the degree of cross-linked proteins. They also suggested that the coat assembly and composition might play a role in the thermal resistance of the spores even if they could not strictly demonstrate it in their study^57^. This could also influence the difference observed here in spore stability between spores produced on plates and in liquid.

When comparing WT PS533 and coatless PS4150 spores produced on Spizizen medium, we could notice that this medium led to more stable spores for the coat deficient mutant than did 2*SG medium (data not shown). However, the mutant underwent the germination process during the temperature increase while the wild type did not (see Fig. 4). This result suggests that the coat might have a role in the stability of spores exposed to a progressive temperature raise. Indeed, the inner membrane of coat deficient spores is estimated to be 4 times more permeable than the one of the wild-type^5^. This was recently confirmed by Knudsen and co-workers^58^, who showed that all coat mutations had a strong effect on water exchange within bacterial spores and especially on *cotE* and *gerE* mutations. To conclude regarding spore stability, we could assume that the discrepancies with respect to spontaneous germination might be related to spore permeability through some variations of coat proteins proportion and cross-linking for the spore produced in liquid medium, or through the complete absence of the coat for PS4150. Unfortunately, to date most studies have been performed on spores produced on agar plates and no study compared spore permeation properties between liquid and agar produced spores. Concerning the DPA-less mutant (see Fig. 5), it was very stable as function of temperature, as it lacks the large DPA depot which activates the enzymes responsible of cortex lysis when released by the spore, and the key enzymes SleB mandatory for germination^35^.

### Role of hydration

The dynamics at the sub-nanosecond level has been largely related to the local environment of proteins and internal motions. It is established that proteins evolve with hydration to reach full mobility around 0.4 *h*, where the proteins are approximately covered by one to two layers of water molecules^50^. Earlier, some authors argued that a low core hydration could be responsible for the extreme resistance of bacterial spores^59,60^, but this would be at odds with the study of Sunde *et al*.^5^ which suggests that the proteins in the core were supposed to be hydrated at 0.6 g H_2_O/g. Water in bacterial spores thus mostly behaves as bulk water, the slow fraction being essentially the ions’ and macromolecules’ solvating water, as observed in binary protein-water systems^14^. The assumption that water might be not homogeneously distributed within the core rendering the rest of the cells extremely compact should be considered, however, our results showed that macromolecules from the core are at least sufficiently hydrated to act like a binary protein-water system at hydration >0.4 *h*.

In general, such a level of hydration should not reduce the local dynamics measured by neutron scattering on the sub-nanosecond timescale and the force constants observed in our study seem to confirm that spore macromolecules behave as fully hydrated components. Indeed their force constants are of the same order as those found in fully hydrated membrane fragments, where no confinement effects are expected^57^. It is, however, very important to note that the time scales accessible by experiments performed with fluorescent probes^14^, by NMR^5^ or neutron scattering vary by several orders of magnitude from micro- or nanoseconds to picoseconds (in the case of neutron scattering). An important observation of these experiments is that *Bacillus subtilis* spores present very similar MSDs at 310 K and effective force constants to the one observed for a mesophilic bacterium such as *E. coli*, in between 0.25 and 0.4 N/m in our case (see Table 3) against 0.39 N/m for the latter one^22^. Moreover, the force constants of FB122 DPA less and coatless PS4150 mutants are almost comparable to the wild type in the low Q region (see Table 3) showing that core resilience is close to the one of the “free” part of the spore (i.e. cortex and coat). It looks counterintuitive as the resistance of spores has been related by several authors to a lower mobility of cores’ components, and we could have expected higher effective force constants. However, the relative immobility of proteins in the core reported by Cowan and co-workers^14^ was observed at the micro- to nanosecond timescales, what cannot be directly related to our approach and should better reflect diffusion of macromolecules in the core. In the present case, the global samples’ dynamics is extremely similar to that of typical biological cells, where intracellular water solvating biomolecules and ions has a mobility that is slightly reduced^22^. Nevertheless, the force constant of a fully germinated sample was found to be very low (~0.10 N.m^−1^), even if somewhat comparable to binary protein water system such as hemoglobin or red blood cells (≈0.16 N.m^−1^)^61^, but the variation is substantial as compared to dormant spores in similar conditions. This leads to the conclusion that proteome within spores is more resilient than in germinated spores, but is not especially high as compared to various hydrated biological models. The k’ values obtained here also showed that the extreme resistance of bacterial spores is not correlated to an adaptation of the macromolecules to high temperatures as it has been observed for the proteins of thermophile bacteria^22^.

Our experiments tend thus to show that the whole spore can be considered as in a fully hydrated state. The previous assumptions, that spore resistance might be related to a glassy state in the core^10,11^ were concluded from DSC and NMR experiments and have been re-evaluated by several authors since^5,12,62^. However, it has been suggested that the CaDPa itself might be in an amorphous solid like state, but might be associated with some molecules of water^62^. Recently, Tros *et al*. used dielectric spectroscopy to compare directly the water dynamics in *E. coli*, yeast and *B. subtilis* and found very similar characteristics showing that most of the water in spores had exactly the same orientational mobility as bulk water^16^. Experiments in binary protein/water-solute systems performed with neutron scattering showed that proteins trapped in a glassy water state as induced, for instance, by trehalose remained harmonic even at temperatures above 273 K presenting very low MSDs and high force constants. For instance, trehalose coated myoglobin has MSDs about seven times lower than hydrated myoglobin, which are close to those of pure trehalose^63^. Interfacial and intracellular water is indeed directly involved in the formation of amorphous matrices, with glass-like structural and dynamical properties. Its dynamics is then slowed down and could influence the dynamics of the atoms to which it is bound. Even for the coatless PS4150 mutant hydrated at 0.4 *h*, no clues of such glassy state could be drawn from our data, with values of k’ typical of the one encountered for binary protein/water systems at the same hydration level. Our finding confirms the most recent theories and shows that the spores exhibit a dynamical behavior that is far from what could have been observed for a glassy state matrix and might rather be considered as a gel state.

### Internal dynamics

The internal dynamics of the proteome of all samples can be essentially observed at high Q corresponding to small amplitudes. All MSDs lie below 2 Å^2^ and mostly even closer to 1 Å^2^. Again these values are very close to what was found in bacteria by Tehei *et al*.^22^ within the same range of temperatures.

Comparing the motional amplitudes <u²>, some differences appeared which permit to sort the samples according to their relative flexibility at high and low Q (see Table 2):

at low Q: < u^2^ > _PS4150-coatless_ ≈ <u^2^ > _PS533_ **<<** < u^2^ > _FB113ger_ **<<** < u^2^ > _FB122-DPAless_ **<<** <u^2^ > _PS533ger_

at high Q: < u^2^ > _PS4150-coatless_ **<<** <u^2^ > _PS533_ ≈ < u^2^ > _FB122-DPA less_ ≈ <u^2^ > _FB113ger_ **<<** u^2^ > _PS533ger_

Core motions should be better resolved in coatless PS4150 because of the lack of most of the proteinaceous coat layer (roughly 40% of total spore volume and 50–80% of spore’s total proteins^1^). As the cortex is composed of loosely cross-linked peptidoglycan as compared to the coat that consists of dense layers of highly cross-linked proteins^1^ we can assume that the more constrained motions in the coatless PS4150 spores originated from the core rather than from the remaining cortex. Thus, the lower MSDs at high Q for the mutant might indicate that motion of H atoms belonging to core’s macromolecules or small molecules’ are limited by confinement. However, the resilience k’ of coatless PS4150 was very similar to the WT PS533 (Table 3), but also to typical biological system in a fully hydrated state such as *E. coli* or binary proteins - water solution as already discussed earlier.

A model that could allow the existence of fully hydrated proteins, which are at the same time highly resistant to thermal denaturation, could consist in their confinement. Some authors showed that proteins in small hydrophilic cages are more resistant than in bulk water^64^. The CaDPA plays an important role in the stability and resistance of bacterial spores to various stresses such as heat, desiccation, UV radiation and chemicals. It contributes to the reduction of the core water content, but it seems that the latter does not explain the extreme resistance of bacterial spores and that the protective effect of DPA accumulation is not related to this feature^13^. On the contrary, living organisms are able to compensate for extreme environmental conditions and hence rescue proteins from denaturation by using osmolytes^65–67^. Organic osmolytes are accumulated under anhydrobiotic, thermal, or pressure stresses. Among the osmolytes are amino acids, sugars, methylamines such as trimethylamine-N-oxide (TMAO), and urea. Hyperthermophiles such as *Thermococcales* are known to accumulate sugar conjugates such as di-myo-inositol phosphate as a function of thermal stress and mannosyl-glycerate as a function of salinity stress^68^. It remains a challenge to check whether such mechanism could also play a role in spores and explain their resistance or if DPA could have a similar significance here.

Indeed, for the DPA-less FB122 mutant, MSDs at low Q are ~1.8 times higher than MSDs of the wild type. Two hypotheses could be formulated to explain such behavior: (i) one of the roles of the DPA may be to stabilize the core through a reduction of diffusive processes in this compartment, and/or (ii) DPA itself is largely immobilized and leads to a reduction of the MSDs observed for the wild-type. The increase in MSDs due to germination was only observed at low Q and suggests that it refers to larger diffusive movements, probably related to the release of small molecules like ions and DPA, and to the lysis of macromolecules such as peptidoglycan from the cortex or SASP proteins in the core that arise within this process. The MSDs of fully germinated spores at low Q were even higher than the dynamics of DPA-less spores suggesting that the core’s compact structure is also responsible for the restriction of larger amplitude motions within it. On the contrary, at high Q the local dynamics was quite similar for the wild type, the DPA-less spores, the partially germinated spores, and even for the fully germinated spores as observed through the MSDs’ ratio lying within 1.12 and 1.31 (see Table 2).

It means that DPA and compact structures of the core of dormant spores both participate to the reduction of larger amplitude motions, but have almost no effect on smaller amplitude motions of the core components observable at high Q.

The fact that partially germinated spores FB113ger had lower MSDs than DPA-less FB122 at low Q, closer to the wild-type ones, is a clue that DPA accumulation during sporulation probably plays a role in the singular organization of the core that is poorly affected even after the release of the Ca-DPA as proposed earlier^13,14^. However, our results revealed that if no influence of Ca-DPA on core’s protein mobility was observed at the second-time scale, some were noted in the present study at the sub-nanosecond time-scale, and to a larger extent for spores that do not accumulate DPA during sporulation (FB122). The role of the accumulation of DPA far upon its limits of solubility in the core could result in an “overcrowding” of the core, leading to the confinement of core components and perhaps of core compartmentalization, restricting the rotation and self-diffusion of macromolecules such as proteins as proposed earlier by Sunde *et al*.^5^. This particular feature could provide a protective environment in the core to physical stresses such as wet heat.

## Conclusion

In the present study, we used elastic incoherent neutron scattering to investigate molecular dynamics of *Bacillus subtilis* spores in their wild-type form and of mutants, lacking some essential parts of the structure. Such strategy permitted to highlight the motions within specific parts of the spores excluding others. Neutrons are particularly sensitive to movements of hydrogen atoms and of subgroups to which they are bound. We were able to disentangle small or large motional amplitudes through the distinction of ranges of transferred momenta Q. Our results permitted to refute the idea that water is in a glassy state within the spores, because we found its dynamical characteristics very close to fully hydrated biological systems. Therefore, we cannot support the idea that such configuration is contributing to the exceptional resistance of such spores. In contrary, according to our results DPA accumulation during sporulation could be key to explain the reduction in core mobility, mainly through the reduction of motions of larger amplitude.

We found furthermore, that the media, on which the spores were grown, influenced the molecular dynamics, but also the stability of the spores through the observation of a spontaneous germination process.

## Supplementary information

Supplementary information.
