# Supplementary material for: The molecular dynamics of bacterial spore and the role of calcium dipicolinate in core properties at the sub-nanosecond time-scale
Source: Sci Rep. 2020 May 19;10:8265. doi: 10.1038/s41598-020-65093-y (PMC7237433; doi:10.1038/s41598-020-65093-y)
Supplement: Supplementary file 1 — Supplementary information. [file 41598_2020_65093_MOESM1_ESM.docx]

**Electronic supplementary information**

**PROTON DYNAMICS IN BACTERIAL SPORES, A NEUTRON SCATTERING INVESTIGATION**

Alexandre COLAS de la NOUE, Francesca NATALI, Fatima FEKRAHOUI, Patrick GERVAIS, Nicolas MARTINEZ, Jean-Marie PERRIER-CORNET, Judith PETERS

**Figure S1:** Temperature dependence of the MSD extracted from the low (left side) and high Q range (right side) of wild spores in H_2_O or D_2_O solutions grown in different sporulation media: 2*SG liquid (in blue), 2*SG agar (in red) and Spz agar in H_2_O (in open green circles) and D_2_O (in filled green circles). The straight lines are linear fits to extract the effective force constants in different temperature domains (see table 3).

**Figure S2:** Ln(I/I_0_) plotted versus Q² of the wild-type spores PS533 grown in 2*SG liquid and probed by neutron scattering in D2O phosphate buffer solution. The straight lines are linear fits to extract <u²> for each temperature at Low Q and High Q range. The important breakdown in linearity between fits at low Q and high Q above 306K illustrates the observation of germination that is only visible at low Q.
